# Inhibition of BDNF-AS Provides Neuroprotection for Retinal Ganglion Cells against Ischemic Injury

**DOI:** 10.1371/journal.pone.0164941

**Published:** 2016-12-09

**Authors:** Lifang Xu, Ziyin Zhang, Tianhua Xie, Xiaoyang Zhang, Tu Dai

**Affiliations:** 1 Department of Ophthalmology, Wuxi People’s Hospital, Wuxi, Jiangsu, China; 2 Department of Hepatobiliary, Wuxi No.2 People’s Hospital, Wuxi, Jiangsu, China; Rutgers University, UNITED STATES

## Abstract

Background: Brain-derived neurotrophic factor (BDNF) protects retinal ganglion cells against ischemia in ocular degenerative diseases. We aimed to determine the effect of BDNF-AS on the ischemic injury of retinal ganglion cells. Methods: The levels of BDNF and BDNF-AS were measured in retinal ganglion cells subjected to oxygen and glucose deprivation. The lentiviral vectors were constructed to either overexpress or knock out BDNF-AS. The luciferase reporter gene assay was used to determine whether BDNF-AS could target its seed sequence on BDNF mRNA. The methyl thiazolyl tetrazolium assay was used to determine cell viability, and TUNEL staining was used for cell apoptosis. Results: The levels of BDNF-AS were negatively correlated with BDNF in ischemic retinal ganglion cells. BDNF-AS directly targeted its complementary sequences on BDNF mRNA. BDNF-AS regulated the expression of BDNF and its related genes in retinal ganglion cells. Down-regulation of BDNF-AS increased cell viability and decreased the number of TUNEL-positive retinal ganglion cells under oxygen and glucose deprivation conditions. Conclusion: Inhibition of BDNF-AS protected retinal ganglion cells against ischemia by increasing the levels of BDNF.

## 1. Introduction

The interruption of blood flow to the retina results in the progressive loss of retinal ganglion cells (RGCs)[1] and plays the fundamental role in many ocular degenerative diseases such as diabetic retinopathy and glaucoma[2]. RGCs transmit visual information from the retina to the brain in the form of action potentials[3]. As a type of neuron, it is difficult for regenerate lost RGCs, which limits the recovery of visual function. Thus, early neuroprotection may extend the therapeutic window in ischemic conditions.

When exposed to ischemia, cells usually trigger the endogenous protection process. As for RGCs, endogenous brain-derived neurotrophic factor (BDNF) is abundant when exposed to exogenous damage[4].BDNF is a member of the neurotrophin family, which is richly expressed during embryonic development and contributes greatly to the development of the nervous system by participating in axonal and dendritic growth. Knockout of the BDNF gene causes thanatophoric dysplasia. BDNF promotes RGC axon branching during retinocollicular/tectal map formation[5]. In adults, BDNF is expressed at relatively low levels while it regulates synaptic transmission and plasticity. As for ocular diseases, administration of BDNF restores visual function. Two weeks of BDNF treatment could maintain long-term central vision in an optic nerve trauma model[6]. For traumatic optic trophy, transplantation of mesenchymal stem cells (MSCs) decreases RGC apoptosis by secreting BDNF[7]. However, endogenous BDNF was persistently down-regulated in the ischemic retina[8].

BDNF antisense RNA (BDNF-AS, also known as BDNF-OS) inhibits the expression of BDNF *in vitro* and *in vivo*[9]. BDNF-AS is one type of long non-coding RNAs (lncRNAs) transcribed by RNA polymerase II without an open reading frame[9]. Compared with other non-coding RNAs (e.g., microRNAs and small interfering RNAs), lncRNAs are defined as longer than 200 nucleotides in length. lncRNAs regulate physiological and pathophysiological processes by modulating the stability and nuclear retention of their target mRNAs[10]. In the present study, we sought to determine the role of BDNF-AS in ischemic insults to RGCs.

## 2. Methods and Materials

### 2.1 Animals

Either 3-month-old male (6 for mating) or 42 newborn C57BL/6 mice were used in the present study. All experiments were performed according to the National Institutes of Health Guide for the Care and Use of Laboratory Animals and approved by the Institutional Animal Care and Use Committee of Nanjing Medical University. The animals were housed under controlled environmental conditions with an ambient temperature of 25°C, relative humidity of 65%, and 12/12-h light-dark cycle. Food and water were provided *ad libitum*. All efforts were made to minimize the number of animals and their suffering.

### 2.2 Cell culture

Primary retinal ganglion cells (RGCs) were isolated according to the two-step immunopanning method as previously reported[11]. After the deep anesthesia by isoflurane, the animals were sacrificed by CO_2_ asphyxiation followed by decapitation. Then the whole retina was isolated and incubated in a papain solution (16.5U/mL, Sigma-Aldrich, US) for 30 min. Next, macrophages and endothelial cells were removed from the cell suspension by panning with an antimacrophage antiserum (Accurate Chemical, Westbury, NY). RGCs were specifically bound to the panning plates containing anti-Thy1.1 antibody (2 μg/ml; Chemicon, US) and released by trypsin treatment. RGCs were grown in serum-free basal medium (Neurobasal/B27 medium; Invitrogen, US).

### 2.3 Oxygen and glucose deprivation (OGD)

RGCs were deprived of oxygen using an anaerobic chamber (0% O_2_, 5% CO_2_ and 95% N_2_) and glucose-and sodium pyruvate-free medium at 37°Cfor different time courses based on the experimental design. After oxygen and glucose deprivation (OGD), the culture medium was exchanged for fresh medium, and the PGCs were further incubated for another 24 h in a 5% CO_2_ atmosphere. Parallel cultures were exposed to oxygenated medium in a normoxic incubator (5% CO_2_) at 37°C.

### 2.4 Real-time PCR

Total RNA was isolated from cells and tissues using the RNeasy Mini Kit (Qiagen, German) and the reverse transcription was performed using the First-strand cDNA synthesis kit (Promega, US). The amplification and data acquisition were performed on a real-time PCR system (Agilent MX3000P, US) using SYBR green PCR Master Mix (Qiagen). The primers used have been reported previously[9]. The conditions were pre-denaturation at 95°C for 15 min followed by 40 cycles at 95°C for 15 s, 60°C for 1 min and 72°C for 1 min and a final dissociation stage at 95°C for 15 s and 55°C for 1 min. All samples were analyzed in triplicate in 3 independent experiments. Relative quantification of mRNA expression was determined using the comparative Ct method.

### 2.5 Western blot

Total protein was obtained using RIPA buffer with cocktail inhibitors (Cell Signaling Tech.). Protein concentration was measured using a BCA kit (Pierce, USA). Equal amounts of protein were separated on a15% gel and then transferred to 0.22μm PVDF membranes (AmerSham, USA). The membranes were blocked in 5% bovine serum albumin (BSA) in Tris-buffered saline with Tween 20 buffer (TBST) for 2 h and then incubated overnight at 4°C with the following primary antibodies: anti-Myc (1:1000, Cell Signaling Tech.) and anti-β-actin (1:1000, Cell Signaling Tech.). Then, the membranes were washed 3 times with TBST and incubated with horseradish peroxidase-conjugated secondary antibody (goat anti-rabbit IgG, 1:5000 or goat anti-mouse IgG, 1:5000,Cell Signaling Tech.) for 1 hatroom temperature. Blots were developed using a chemiluminescence kit (Pierce) and exposed to X-ray film. The bands on the film were scanned and analyzed with Quality One software (Bio-Rad).

### 2.6 Enzyme-linked immunosorbent assay (ELISA)kits

The concentrations of BDNF, IL-2, IL-6 and TNF-α were determined using quantitative sandwich enzyme immunoassays with ELISA kits (R&D, USA). The procedure was conducted according to the manufacturer’s recommendation. The inter-assay coefficient of variability of the cytokine assays was less than 10%, and the intra-assay coefficient of variability was less than 10% across the concentration range.

### 2.7 Construction of lentiviral vectors

The gene fragment (BDNF-AS) was subcloned into pcDNA3to formpcDNA3-BDNF-AS. The pcDNA3-BDNF-AS and shBDNF-AS DNA were subcloned into the lentiviral vector pLVX-IRWS-ZsGreen1 due to its high efficiency. Lentiviral vectors were produced and titrated according to a previous report[12]. The lentiviral vectors were stored at -80°C until use.

### 2.8Luciferase assay

The BDNF gene fragment was subcloned into the pMIR-REPORT vector (Ambion, US) to develop the pMIR-BDNF-3′-UTR luciferase vector. Cells at a density of 2×10^5^ per well were seeded into 24-well plates and cultured overnight. The cells were co-transfected with 1.0 μg of either pMIR-BDNF-3′-UTR or pMIR-REPORT vector, 5.0 μg of pcDNA3-BDNF-AS and 50 ng of pRL-TK vector as an internal control using Lipofectamine 2000 reagent (Invitrogen). The cells were harvested 24 h post-transfection and used to determine the luciferase activity by a dual luciferase reporter assay kit (Promega, USA) [13].

### 2.9 3-(4,5-dimethylthiazol-2-yl)-2,5-diphenyltetrazolium bromide (MTT) assay

Cells at a density of 5×10^3^ per well were seeded into 96-well plates and infected with lentiviral vectors24 h later. After another 24 h, 20 μl of 5 mg/mL MTT (dimethyl thiazolyl diphenyl tetrazolium, Sigma-Aldrich) was added to each well, and the cells were incubated for 4 h in a humidified incubator. The supernatant was discarded, and 200 μL of DMSO was added. The optical density was measured at 490 nm.

### 2.10 TUNEL staining

TUNEL staining was performed using a kit (InSitu Cell Death Detection Kit, POD; Roche, USA) according to the manufacturer’s instructions. In brief, the RGCs were fixed for 20 min with 4% paraformaldehyde in PBS and pretreated with 3% H_2_O_2_ in methanol and 0.1% TritonX-100. The TdT enzyme and nucleotide mix were then added, and the slides were maintained for 60 min at 37°C followed by three washes with PBS (pH 7.4). Then, the horseradishperoxidase (POD) was added, and the slides were incubated for another 30 min at 37°C. After three washes with PBS, diaminobenzidine (DAB) was added and incubated for 10 min at room temperature. The slides were mounted on a glass coverslip, and the positive cells were analyzed under light microscope.

### 2.11 Statistics

Parameter data were expressed as the mean±standard deviation and were analyzed with an unpaired *t*-test and analysis of variance (ANOVA) followed by a post-hoc *t*-test. Differences between proportions were assessed by the χ^2^ test. The survival analysis was conducted using the Kaplan-Meier method. All the analyses were conducted using SPSS13.0 software. Statistical significance was defined as *P*<0.05.

## 3. Results

### 3.1 Inverse trend of BDNF-AS and BDNF in RGCs *in vitro* after ischemia

**Fig 1:**
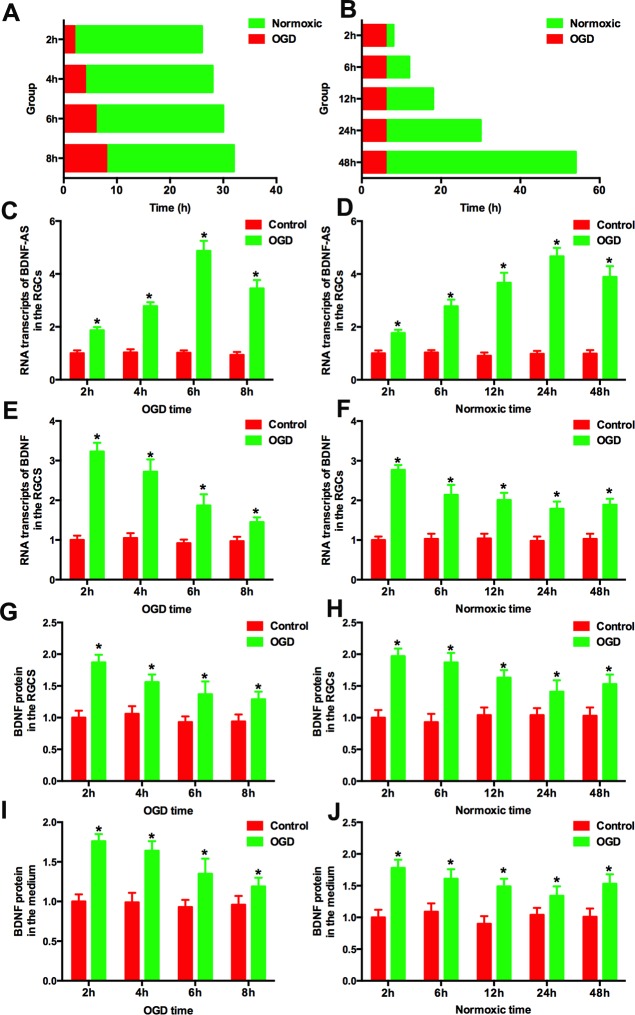
Elevation of BDNF-AS in ischemic RGCs. (A) RGCs were subjected to ischemia for different lengths of time following 24 h of normoxic culture. (B) RGCs were exposed to ischemia for6 h and harvested at different times. (C) BDNF-AS was increased in ischemic RGCs. The peak levels were achieved after 6 h of ischemia. (D) Elevation of BDNF-AS was observed at different time points after 6 h of ischemia. (E) BDNF mRNA was increased in the ischemic group while a trend was negatively correlated with BDNF-AS. (F) BDNF mRNA was inversely related with BDNF-AS at different time points after 6 h of ischemia. BDNF protein levels in the RGCs (G) and medium (I) were correlated with BDNF mRNA levels. After 6 h of ischemia, the RGCs (H) and their medium (J) were harvested at different time points, and BDNF protein levels maintained a similar trend with mRNA expression. *P*<0.05 was considered significantly different; * vs control group.

Primary RGCs were cultured in the dish. Firstly, the RGCs were exposed to OGD over different courses (2 h, 4 h, 6 h and 8 h) followed by 24 h normoxic culture (Fig 1A). The medium and cells were harvested for analysis. RT-PCR was used to measure the levels of BDNF-AS and BDNF mRNA of cells. The concentration of BDNF in the medium and cells was determined using ELISA. As shown in Fig 1C, BDNF-AS was significantly increased by OGD exposure when compared to the control group. The level of BDNF-AS was significantly increased after 2 h of OGD treatment (1.87±0.12 vs 1.0±0.11, n = 6, *P*<0.05) and peaked after 6 h (4.87±0.38 vs 1.02±0.09, n = 6, *P*<0.05). There was a slight decrease after 8 h of OGD treatment (3.45±0.32 vs 0.94±0.11, n = 6, *P*<0.05) compared with the 6-h OGD treatment partially due to the decreased cell number after 8 h of OGD exposure. However, the level of BDNF mRNA showed an opposing trend (Fig 1E, S4 Table). BDNF mRNA levels in RGCs decreased after 2 h of OGD treatment, and the trend was enhanced as the OGD incubation time was extended. A similar enhanced trend was observed with regard to the concentration of BDNF protein derived from the cells (Fig 1G, S5 Table) and medium (Fig 1I, S8 Table).

Then, we harvested the cells or medium at different time points (2 h, 6 h, 12 h, 24 h and 48 h) after 6 h of OGD exposure (Fig 1B). The level of BDNF-AS was significantly increased as early as 2 h after the addition of OGD. The peak of BDNF-AS was 24 h after OGD treatment, and the elevated levels of BDNF-AS remained at least 48 h after OGD treatment (Fig 1D, S2 Table). The levels of BDNF mRNA were significantly decreased 12 h after OGD treatment (Fig 1F, S4 Table). Compared to 24 h after OGD treatment, there was a slight but significant increase in BDNF mRNA levels at 48 h after OGD treatment. The BDNF protein derived from the cells (Fig 1H, S6 Table) and medium (Fig 1J, S8 Table) showed the similar trend as the mRNA.

### 3.2 BDNF-AS inhibits the expression of BDNF

**Fig 2:**
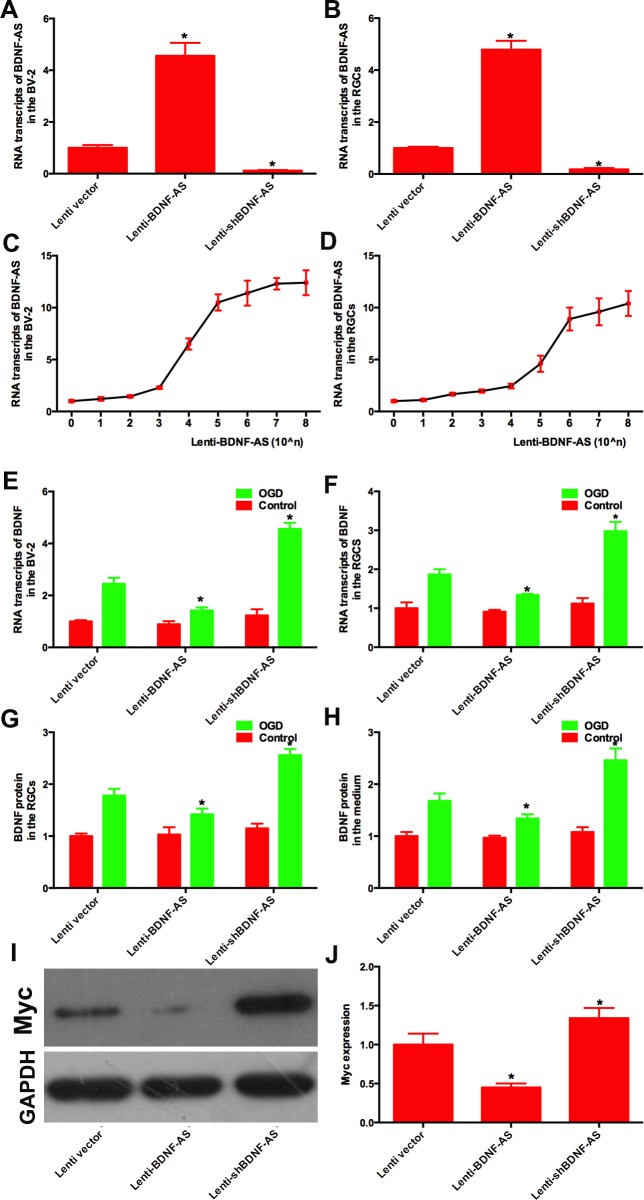
BDNF-AS reduced the expression of BDNF. (A) In BV-2 cells and (B) RGCs, lenti-BDNF-AS increased the level of BDNF-AS while the lenti-shBDNF-AS reduced the level of BDNF-AS. Lenti-BDNF-AS increased the levels of BDNF-AS in the dose-dependent manner in BV-2 cells (C) and RGCs (D). (E) In BV-2 cells under normal conditions, BDNF mRNA levels were not significantly changed by either lenti-BDNF-AS or lenti-shBDNF-AS while under OGD conditions, BDNF mRNA levels were decreased by lenti-BDNF-AS and increased by lenti-shBDNF-AS. (F) In normal RGCs, BDNF mRNA levels were also unchanged by either lenti-BDNF-AS or lenti-shBDNF-AS. When RGCs were subjected to 6 h of ischemia, BDNF mRNA levels were decreased by lenti-BDNF-AS and increased by lenti-shBDNF-AS. The total protein was derived from (H) RGCs and (I) the culture medium. The BDNF protein was measured by ELISA and showed a similar trend as observed with mRNA expression. (J) The BV-2 cell line expressing Myc-tagged BDNF. Lenti-BDNF-AS reduced Myc expression while lenti-shBDNF-AS increased this expression. (K) represents the summarized data of the western blots. *P*<0.05 was considered significantly different; * vs control group.

The gene fragment of BDNF-AS was cloned into a lentiviral vector and termed Lenti-BDNF-AS. The level of BDNF-AS mRNA was significantly increased in BV-2 cells transduced with Lenti-BDNF-AS (Fig 2A). BDNF-AS mRNA was increased in the dose-dependent manner (Fig 2C). As for primary RGCs, Lenti-BDNF-AS significantly increased the level of BDNF-AS mRNA (Fig 2B) in a dose-dependent manner (Fig 2D), which decreased the level of BDNF mRNA (Fig 2F, S10 Table). We then measured BDNF protein levels using ELISA and found that Lenti-BDNF-AS decreased the level of BDNF protein in the RGCs (Fig 2H, S12 Table) and medium (Fig 2I).

To further understand the relationship between BDNF-AS and BDNF, we constructed BDNF-AS siRNA and cloned the shRNA into a lentiviral vector termed Lenti-shBDNF-AS as described in a previous report. The BDNF-AS mRNA level was significantly decreased by Lenti-shBDNF-AS in BV-2 cells (Fig 2A) and primary RGCs (Fig 2B). Twenty-four hours after transduction of either Lenti-shBDNF-AS or control vector, primary RGCs were exposed to 6 h of OGD followed by 24 h of culture. The BDNF mRNA level was significantly increased by Lenti-shBDNF-AS compared with the control vector (Fig 2E and 2F, S9 and S10 Tables). In normal RGCs transduced with Lenti-shBDNF-AS, the BDNF protein was also significantly increased (Fig 2H and 2I, S12 Table).

We then constructed a cell line derived from BV-2 cells that overexpresses BDNF with a Myc tag. When this new cell line was transduced with Lenti-BDNF-AS, Myc was significantly decreased compared with the control group (Fig 2J and 2K). However, the Myc protein levels were higher in the cells transduced with Lenti-shBDNF-AS (Fig 2J and 2K).

### 3.3 Inhibition of BDNF-AS promotes the survive of RGCs against ischemia

**Fig 3:**
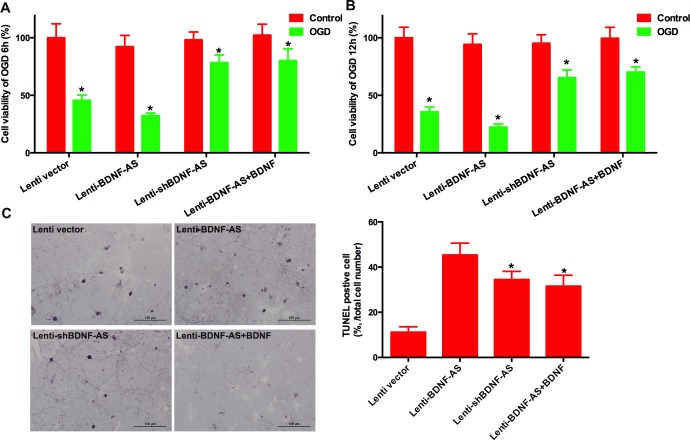
Inhibition of BDNF-AS protected RGCs against ischemia. The MTT method was used to determine cell viability. (A) Lenti-BDNF-AS significantly decreased cell viability while the lenti-shBDNF-AS reversed this trend. The effect of lenti-BDNF-AS on cell viability could be reversed by exogenous BDNF protein. (B) When RGCs were exposed to 12 hof OGD, similar results were observed. (C) The TUNEL method was used to measure apoptosis. The number of TUNEL-positive cells was increased by lenti-BDNF-AS but decreased by lenti-shBDNF-AS compared with the empty vector. The effect of lenti-BDNF-AS was also blocked by exogenous BDNF protein treatment. Scale bar, 100μm; *P*<0.05 was considered significantly different; * vs control group.

Cell viability measured by the MTT method showed no significant difference among normal primary RGCs transduced with control vector, Lenti-BDNF-AS or Lenti-shBDNF-AS (Fig 3A, S13 Table). However, when primary RGCs were exposed to 6 h of OGD, Lenti-BDNF-AS decreased cell viability while Lenti-shBDNF-AS restored cell viability (Fig 3A, S13 Table). Then, the cell line was transduced with different lentiviral vectors and exposed to 12 h of OGD. Consistent with primary RGCs, Lenti-BDNF-AS decreased cell viability while Lenti-shBDNF-AS restored cell viability (Fig 3B, S14 Table).

To reverse the effect of BDNF-AS, primary RGCs were transduced with different lentiviral vectors and then exposed to OGD for 6 h (Fig 3A, S13 Table) or 12 h (Fig 3B, S14 Table) with exogenous BDNF protein in the culture medium. There was no significant difference incell viability among the three groups.

The number of TUNEL-positive cells was significantly increased by Lenti-BDNF-AS and decreased by Lenti-shBDNF-AS (Fig 3C). Adding exogenous BDNF protein blocked the effect of Lenti-BDNF-AS (Fig 3C).

### 3.4 BDNF-AS targets its complementary sequences in BDNF mRNA

**Fig 4:**
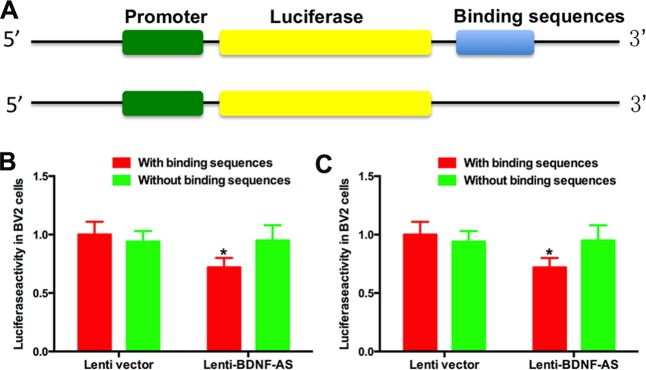
BDNF-AS targets the binding sequence of BDNF mRNA. (A) Luciferase reporter vectors were constructed. (B) In BV-2 cells, the cells were co-transfected with aluciferase reporter vector and Renilla vector and then transduced with lentiviral vectors. When the cells were transfected with the reporter vector containing the binding sequences, lenti-BDNF-AS significantly decreased the luciferase activity. However, in the cells transfected with the reporter vector without the binding sequences, there was no significant difference between the lenti-BDNF-AS and empty vectors. (C)Similar results were observed in RGCs. *P*<0.05 was considered significantly different; * vs control group.

We cloned the complementary sequences of BDNF-AS into the 3’UTR of luciferase as shown in Fig 4A. Then, we co-transfected the vectors into HEK 293T cells. As shown in Fig 4B and 4C, there was no difference of luciferase activity in cells containing the luciferase vector without the target sequence. BNDF-AS inhibited the luciferase activity (Fig 4B) compared with control group while the shBDNF-AS reversed the effect (Fig 4C).

### 3.5 BDNF-AS regulates the expression of BDNF-related cytokines

**Fig 5:**
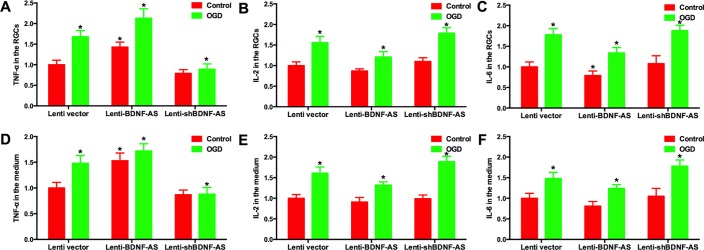
Effect of BDNF-AS on BDNF-related gene expression. RGCs were transduced with different lentiviral vectors and then exposed to OGD. The cells and medium were harvested to measure the protein levels of TNF-α, IL-2 and IL-6 using ELISA. The elevated BDNF-AS increased the levels of TNF-α while lenti-shBDNF-AS decreased the levels in the cells (A) and medium (D). The level of IL-2 in the RGCs (B) and medium (E) was lower in the lenti-BDNF-AS group compared with the lenti-shBDNF-AS group. IL-6 levels in the RGCs (C) and medium (F) were also decreased by lenti-BDNF-AS. *P*<0.05 was considered significantly different; * vs control group.

There is well known cross-talk between BDNF and cytokines, which contributes to the injury and repair of RGCs during damaging conditions. We determined whether BDNF-AS could affect the expression of these cytokines. Primary RGCs were transduced with different lentiviral vectors followed by a 6-hexposure to OGD. The medium and cells were harvested 24 h later. The levels of TNF-α (Fig 5A and 5D, S15 and S16 Tables), IL-2 (Fig 5B and 5E, S16 and S19 Tables) and IL-6 (Fig 5C and 5F, S17 and S20 Tables) were measured by ELISA. The level of TNF-α was positively correlated with BDNF-AS while the levels of IL-2 and IL-6 were negatively correlated with BDNF-AS.

## 4. Discussion

In the present study, we demonstrated that BDNF-AS could inhibit the expression of BDNF in primary RGCs via targeting the complementary sequences in its mRNA. Ischemia-induced BDNF-AS hampered the neuroprotection of endogenous BDNF, and inhibition of BDNF-AS by its specific shRNA promoted the survival of RGCs exposed to OGD. Furthermore, the expression of genes downstream of BDNF was also regulated by BDNF-AS. Our study provided a novel mechanism of endogenous neuroprotection for RGCs against ischemia, which indicated some potential new treatments for ocular degenerative diseases.

BDNF is a major therapeutic target for ocular degenerative diseases that are characterized by ischemia-induced cellular damage in the retina [14]. Delivery of exogenous BDNF reduced the degree of retinal damage and increased the number of surviving ganglion cells under ischemic conditions[15]. However, due to the blood-retinal barrier and blood-cochlear barrier (which have comparable structure to the blood-brain barrier), large molecules such as BDNF have limited access to reach the target cells by systemic administration[16]. Therefore, endogenous BDNF levels play a key role in the survival of RGCs when exposed to ischemic insult[17]. In the present study, we found that the levels of endogenous BDNF were elevated in primary RGCs when exposed to OGD. This finding was consistent with a previous report[18]. In addition, we also found that the BDNF levels decreased as the time of OGD extended. This indicated that some antagonists may inhibit BDNF levels, and removing these antagonists may be a novel treatment. Identification of the antagonist was the first step.

In the previous report, Farzaneh et al. found that BDNF was normally repressed by a conserved non-coding antisense RNA transcript, BDNF-AS[9].BDNF-AS is one example of lncRNAs, which are defined by their nucleotide length (>200).The mouse BDNF-AS transcript has two splice variants containing 1 or 2 exons, but both contain 934 nucleotides complementary to BDNF mRNA. We then determined the levels of BDNF-AS in ischemic RGCs. Our data showed that the levels of BDNF-AS were dramatically increased in RGCs under ischemic conditions. Our data were partially supported by previous studies, which have proven that the expression of lncRNAs were altered in ischemic cells and tissues[19]. However, our study was the first report regarding the level of BDNF-AS in ischemic RGCs.

Furthermore, the level of BDNF-AS was negatively correlated with the level of BDNF mRNA in RGCs under ischemic conditions. This result indicated that BDNF-AS might suppress the expression of BDNF by decreasing BDNF mRNA levels. To prove this point, we altered the levels of BDNF-AS and observed the levels of BDNF mRNA. It was found that elevated BDNF-AS decreased BDNF mRNA levels while knockdown of BDNF-AS increased BDNF mRNA levels. These data were consistent with a previous report[9]. LncRNAs regulate the expression of target genes by binding to complementary sequences[10]. BDNF mRNA contains the complementary sequences of BDNF-AS. However, there lacked direct evidence that BDNF-AS could regulate BDNF by targeting its complementary sequence. In the present study, we used luciferase reporter genes to confirm this hypothesis. It was the first report using a luciferase reporter assay to prove the mechanism of lncRNAs.

BDNF binds to the TrkB receptor to trigger the expression of many genes that play essential roles in neuroprotection against ischemic injury[20]. Cytokines and inflammatory factors such as TNF-α are the main factors. We then determined whether BDNF-AS could regulate the expression of these genes. We initially found that ischemic conditions resulted in alterations of these inflammatory genes. The pro-inflammatory factor TNF-α was positively correlated with BDNF-AS while the anti-inflammatory factors (IL-2 and IL-6) were negatively correlated with BDNF-AS.

Finally, we sought to determine whether inhibition of BDNF-AS could attenuate ischemic injury. The up regulation of BDNF-AS enhanced ischemic injury while knockdown of BDNF-AS by its specific shRNA attenuated ischemic injury in vitro. Our data were indirectly supported by other findings. Mehta et al. found that FosDT, another lncRNA, promoted neuron death under ischemic conditions while knockdown of FosDT significantly ameliorated ischemic injury in vivo[21]. Liu et al. found that UCA1 contributed to cardiomyocyte apoptosis via regulating p27 expression[22].

## 5. Conclusion

In conclusion, we found that BDNF-AS promoted ischemic injury of RGCs via suppression of BDNF and that inhibition of BDNF-AS reduced ischemic injury. Our findings explored the novel mechanism of RGCs under ischemic conditions and may provide an insight into a new treatment strategy for many ocular degenerative diseases.

## Supporting Information

S1 Table Details of Fig 1C. (XLSX)

S2 Table Details of Fig 1E. (XLSX)

S3 Table Details of Fig 1D. (XLSX)

S4 Table Details of Fig 1G. (XLSX)

S5 Table Details of Fig 1H. (XLSX)

S6 Table Details of Fig 1I. (XLSX)

S7 Table Details of Fig 1J. (XLSX)

S8 Table Details of Fig 2E. (XLSX)

S9 Table Details of Fig 2F. (XLSX)

S10 Table Details of Fig 2G. (XLSX)

S11 Table Details of Fig 2H. (XLSX)

S12 Table Details of Fig 2I. (XLSX)

S13 Table Details of Fig 3A. (XLSX)

S14 Table Details of Fig 3B. (XLSX)

S15 Table Details of Fig 5A. (XLSX)

S16 Table Details of Fig 5B. (XLSX)

S17 Table Details of Fig 5C. (XLSX)

S18 Table Details of Fig 5D. (XLSX)

S19 Table Details of Fig 5E. (XLSX)

S20 Table Details of Fig 5F. (XLSX)
